# Enhancing Fixed Partial Denture Pontic Fabrication: An In Vitro Comparative Study of the Digital and Manual Techniques

**DOI:** 10.7759/cureus.65757

**Published:** 2024-07-30

**Authors:** Maaz Vohra, Amrutha Shenoy, Suresh Venugopalan

**Affiliations:** 1 Department of Prosthodontics, Saveetha Dental College and Hospitals, Saveetha Institute of Medical and Technical sciences, Saveetha University, Chennai, IND; 2 Department of Prosthodontics, Saveetha Dental College and Hospitals, Saveetha Institute of Medical and Technical sciences, Saveetha University, chennai, IND

**Keywords:** computer-aided design and computer-aided manufacturing (cad-cam), gingival health, pontic, stereo-microscopy, fixed dental prosthesis

## Abstract

Background

Advancements in computer-aided design (CAD) and computer-aided manufacturing (CAM) technology have significantly improved the accuracy and consistency of producing fixed partial dentures (FPDs) compared to traditional manual methods. However, the fully digital transfer of mock-up morphology to final FPDs is not yet fully explored. Proper pontic design, which avoids direct gingival contact, is essential for maintaining oral hygiene and preventing tissue irritation.

Aim and objectives

This study aims to compare the effectiveness of digital versus manual methods in FPD pontic fabrication, focusing on the trueness of digitally fabricated FPD patterns. Key objectives include assessing thickness, vertical gaps, and anatomical accuracy to determine the advantages of CAD-CAM technologies over traditional techniques.

Materials and methods

In this in vitro study, a total of 45 FPD pontics were fabricated and divided into three groups (15 each): digitally fabricated (using CAD software and CAM systems), manually fabricated (using traditional wax-up techniques), and a control group (typodont teeth). Tooth preparation was performed on a typodont, and impressions were taken to create casts. One cast was scanned and digitally designed, while the other was used for manual fabrication. Outcome assessments included vertical gap measurement using a stereo microscope, thickness evaluation with a digital caliper, and anatomical similarity assessment by independent evaluators. Statistical analysis involved one-way analysis of variance (ANOVA), post hoc Tukey's analysis, and unpaired t-tests using SPSS software version 26.0 (IBM Inc., Armonk, New York). Statistical significance was set at 0.05.

Results

The digital group exhibited lower mean thickness at the incisal (1.92±0.130 mm vs. 2.46±0.219 mm for manual, p=0.000), middle (7.00±0.223 mm vs. 8.88±0.983 mm for manual, p=0.001), and cervical sites (9.06±0.134 mm vs. 10.08±0.454 mm for manual, p=0.000). No significant differences were found between the digital and control groups. No significant differences were observed between digital, manual, and control groups at any site (p=0.688 to 0.997). The digital group demonstrated superior accuracy and consistency compared to the control group (mean value of 1.00±0.00 vs. 2.93±0.798, p=0.000).

Conclusion

CAD-CAM technology greatly improves the precision and consistency of FPD pontic fabrication compared to traditional manual techniques. Digital methods produce thinner pontics with superior anatomical accuracy, although vertical gap measurements are similar across methods. These findings emphasize the benefits of CAD-CAM in enhancing prosthetic outcomes and suggest potential improvements in clinical practices for prosthodontic rehabilitation.

## Introduction

Advances in digital technology, particularly computer-aided design (CAD) and computer-aided manufacturing (CAM), have transformed prosthetic restorations in dentistry. While traditional manual techniques are well-established, they often lack precision and reproducibility. Digital methods, however, are lauded for their potential to enhance accuracy and consistency in fixed partial denture (FPD) production [1].

In prosthodontic rehabilitation for FPDs, clinicians commonly use mock-ups or wax-ups to establish the desired shape before fabricating the final prosthesis. Traditionally, this process involves sharing stone casts and photographs with dental technicians, but accurately transferring these shapes is challenging due to the complex emergence profile and the gingival surface of the pontic [2]. Advances in CAD and CAM technologies have improved the precision of these transfers [3]. An investigator introduced a fully digital workflow that uses intra-oral scanners and implant scan bodies to transfer the shape of implant-supported mock-ups to final restorations [4]. An author combined analog and digital techniques to transfer gingival morphology under the FPD pontic by superimposing scanned three-dimensional (3D) data onto a working cast model. However, these methods often fail to capture the full morphology of the FPD, including the incisal edge and outer tooth shape [5]. A study used a digital workflow to transfer the form of a mock-up but did not fully replicate gingival surfaces and subgingival contours [6]. Despite these advancements, comprehensive digital transfer of the entire mock-up or wax-up morphology to final FPDs remains insufficiently explored, highlighting the need for further research to refine digital prosthetic fabrication techniques [7]. Pontic is a crucial component of fixed or removable partial dentures, mimicking the missing tooth's morphology, and can be adjusted for specific clinical scenarios, such as convex tissue surfaces and narrow occlusal tables. Reducing the buccolingual width of a pontic can minimize interference during eccentric movements, although opinions on the optimal size of the occlusal table vary. Importantly, the pontic should avoid direct contact with the gingival tissue to support oral hygiene and prevent tissue irritation [8]. CAD-CAM technologies offer notable advantages, including efficient vertical manufacturing, material recycling, and the capability to produce complex objects from various materials like polymers, metals, waxes, and ceramics [9]. Traditionally, wax pattern fabrication was time-consuming and operator-dependent, but CAD-CAM technologies now enable more efficient and precise pattern creation [10]. Despite previous studies focusing on interim restorations, the accuracy of CAD-CAM techniques for definitive FPD patterns remains underexplored.

This study aims to evaluate the effectiveness of digital versus manual methods in fabricating fixed partial denture (FPD) pontics, with a focus on the accuracy of digitally produced FPD patterns. The primary objectives include comparing the thickness, vertical gaps, and anatomical accuracy of pontics created by both methods to ascertain the benefits of CAD-CAM technologies over traditional techniques. The null hypothesis posits that there will be no significant differences in thickness, vertical gaps, or anatomical accuracy between digital and manual methods in FPD pontic fabrication.

## Materials and methods

Study design

This in vitro study was conducted with approval from the Institutional Systematic Review Board (Approval number: SRB/SDC/PROSTHO-2105/24/104). A total of 45 fixed partial denture (FPD) pontics were fabricated and divided into three groups, each consisting of 15 pontics. Group 1 comprised digitally fabricated pontics, designed using computer-aided design (CAD) software (Dental Designer 2021; 3Shape, Copenhagen, Denmark). The CAD files were sent to a computer-aided manufacturing (CAM) system (CORiTEC 350i; Imes-iCore, Leibolzgraben, Eiterfeld) for the fabrication of wax FPDs. Group 2 included manually fabricated pontics, created using traditional wax-up techniques. Wax was sculpted to achieve the desired pontic anatomy, ensuring the final pontics met the required specifications. Group 3 served as the control group, representing the desired anatomical characteristics of pontics, using typodont teeth (Nissin, Kyoto, Japan) as the control. The sample size was determined using G*Power analysis (Heinrich-Heine-Universität Düsseldorf, Germany, Version 3.1) based on a prior study, with a significance level of 0.05, a power of 0.85, and an effect size of 0.95 [11].

Sample preparation

**Figure 1:**
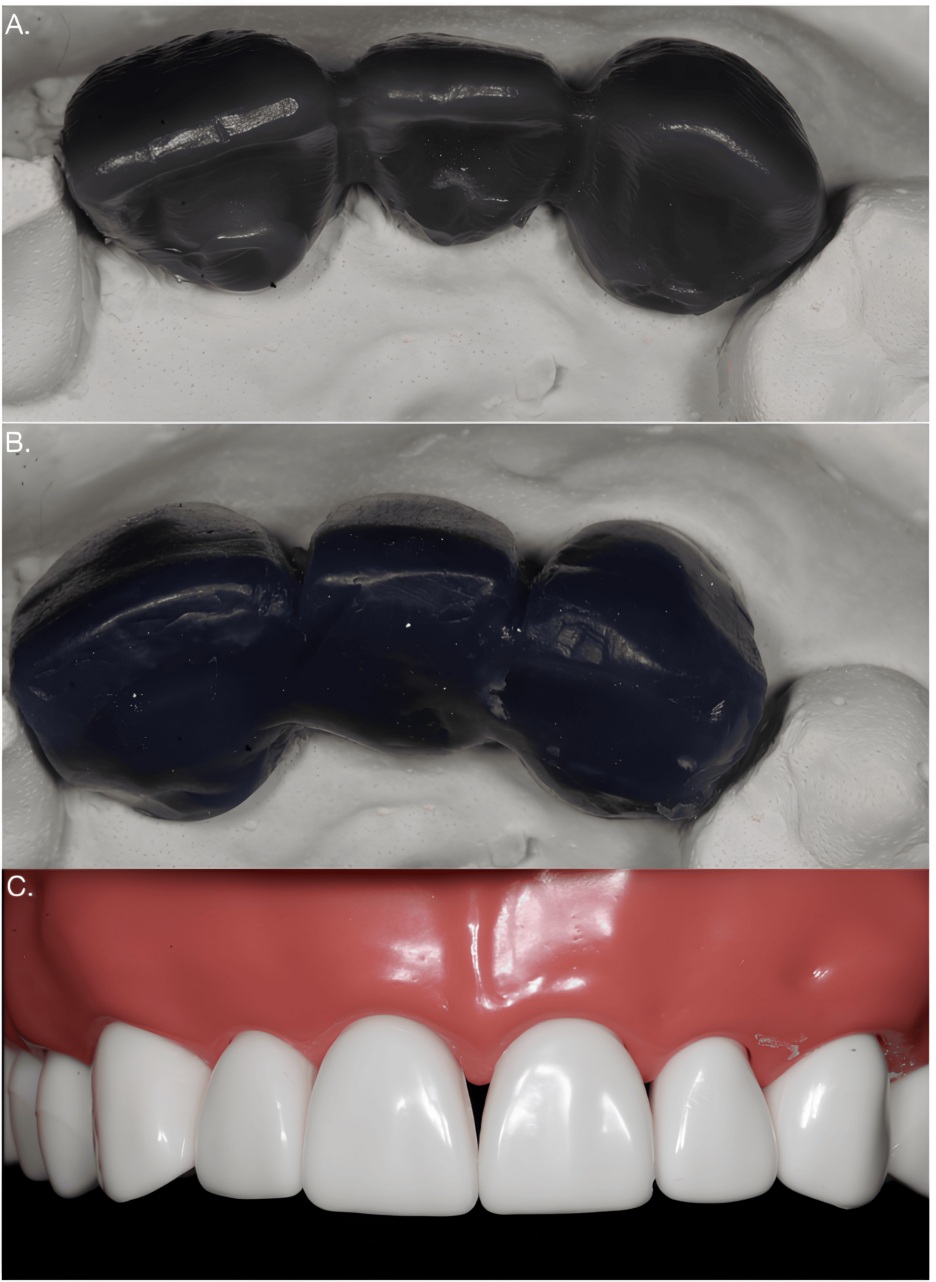
Study design and sample preparation A) Digitally fabricated pontics (group 1); B) Manually fabricated pontics (group 2); C) Typodont teeth (group 3)

Ideal tooth preparation for a metal-ceramic restoration was performed on a typodont (Nissin, Kyoto, Japan), using the maxillary right central incisor and canine as abutments and the maxillary right lateral incisor as a pontic. After the tooth preparation, a single-stage putty wash impression was recorded, and two casts were created using type IV stone (Kalabhai Kalstone, Mumbai, India). One cast was scanned with a laboratory scanner (Trios E4; 3Shape, Copenhagen, Denmark), and digital designing was completed using 3Shape Dental Designer 2021 software (3Shape, Copenhagen, Denmark). The design was then exported as a standard tessellation language (STL) file to a computer-aided manufacturing (CAM) system (Imes-iCore 350i, Leibolzgraben, Eiterfeld), and the pontic was milled from a wax blank (Aidite wax blank, Alphabond Dental, Australia). The second cast was used for manual fabrication (2GM, Mumbai, India). A putty index made from the typodont teeth guided the manual fabrication of the pontic, ensuring the desired anatomy and proper contact with the gingiva were achieved (Figure 1).

Outcome assessment

Vertical Gap Assessment 

**Figure 2:**
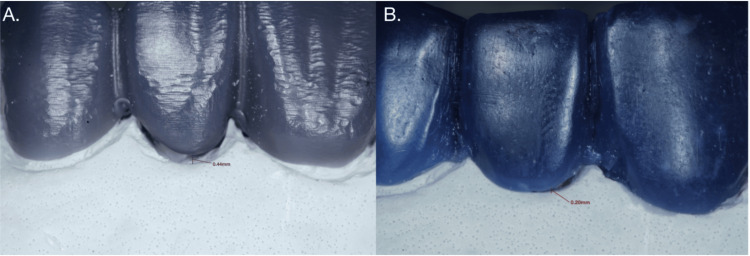
Vertical gap assessment using stereo microscope at a magnification of 10x A) Gap assessment of digitally fabricated pontic; B) Gap assessment of manually fabricated pontic

The vertical gaps between the pontics and the underlying model were assessed using a stereo microscope (Olympus SZ61, Tokyo, Japan) with a magnification range of 10x. The stereo microscope was equipped with a calibrated measurement scale to ensure precise gap measurements. The pontics were positioned on the underlying model, and the vertical gaps were measured at multiple points along the margins. Data were recorded and analyzed to determine the precision of fit for each group (Figure 2).

Thickness Evaluation 

**Figure 3:**
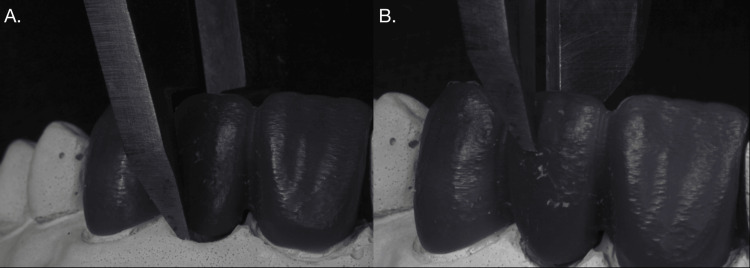
Thickness evaluation of pontic A) Thickness evaluation at cervical third; B) Thickness evaluation at middle third

The thickness of each pontic was measured using a digital caliper (Mitutoyo 500-196-30, Pune, India) with an accuracy of ±0.01 mm. Measurements were taken at three predetermined locations on the pontic: the incisal edge, the middle section, and the cervical region. Each measurement was repeated three times to ensure accuracy and reproducibility, and the mean thickness was calculated for analysis (Figure 3).

Anatomical Similarity Assessment 

The anatomical similarity of the pontics was evaluated by comparing their contour and surface texture to the control group. This assessment was performed by three independent evaluators, each with extensive experience in dental morphology. Evaluation parameters included overall contour, surface smoothness, and anatomical detail, each rated on a scale from one (excellent) to four (poor). Inter-evaluator reliability was ensured by training the evaluators with standardized criteria before the assessment.

This methodological approach ensured a comprehensive evaluation of the pontics' fabrication techniques and their anatomical accuracy. The use of precise instruments and standardized procedures provided reliable and reproducible data for analysis.

Statistical analysis

Data was collected and tabulated using Google Forms (Google, Mountain View, California), and pairwise comparisons of the vertical gap, anatomical form, and thickness values recorded for each group at three different sites were conducted. The differences were analyzed using one-way analysis of variance (ANOVA) and post hoc Tukey's analysis, while the mean vertical gap was assessed using an unpaired t-test, all performed with SPSS software version 26.0 (IBM Inc., Armonk, New York). This analysis was conducted after confirming the normality of the data set, with statistical significance set at 0.05.

## Results

Thickness neasurements

**Figure 4:**
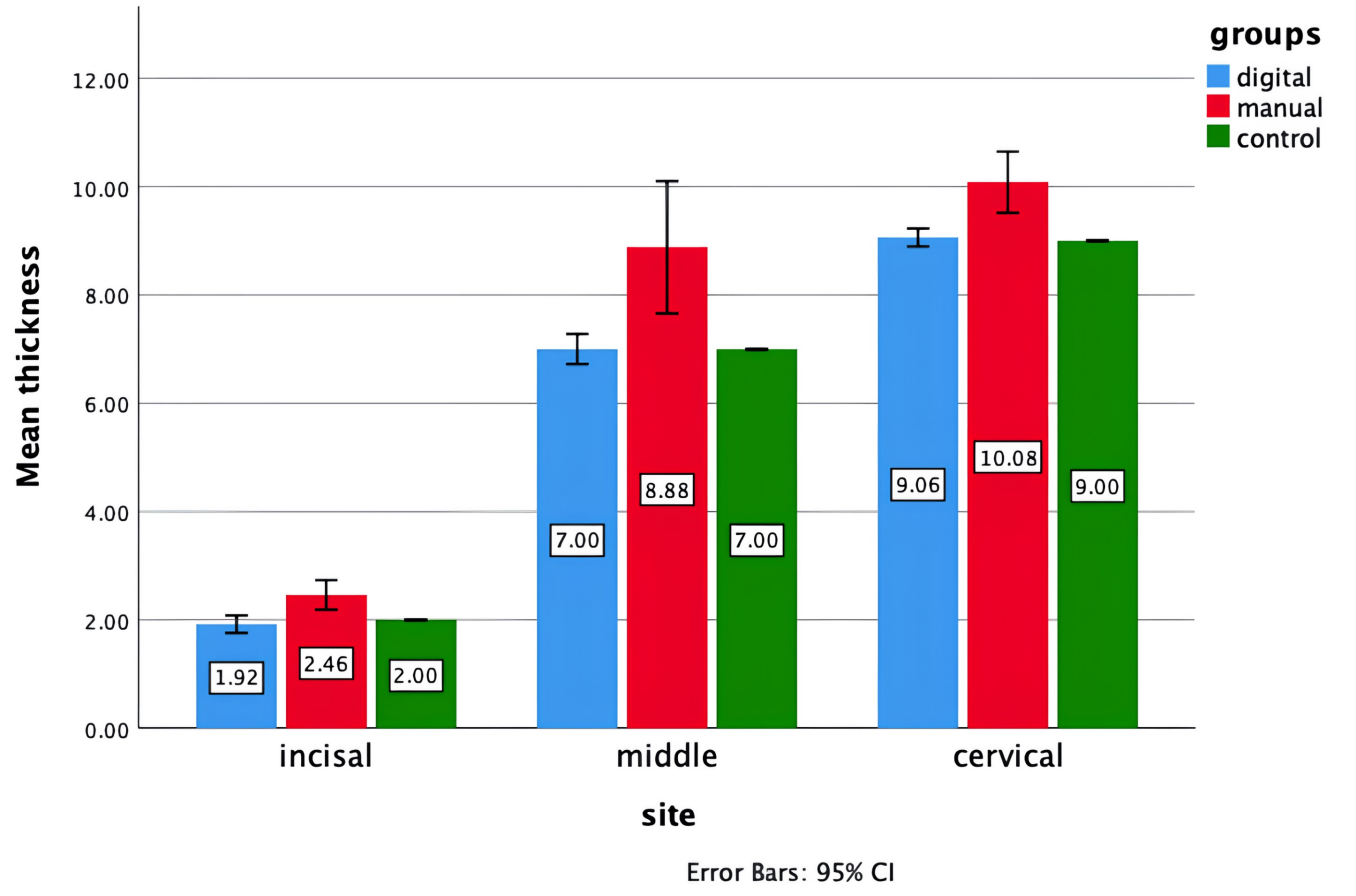
Difference in the thickness of pontics fabricated recorded for each group, per examined site

Table 1 presents the mean thickness values assessed for three groups (digital, manual, and control) at three different sites: incisal, middle, and cervical. At the incisal site, the digital group showed a mean thickness of 1.92±0.130 mm, the manual group had 2.46±0.219 mm, and the control group recorded 2.00 mm. The analysis revealed a statistically significant difference with an F value of 19.600 and a p-value of 0.000. Pairwise comparisons indicated significant differences between the digital and manual groups (mean difference=-0.540, p=0.000) and between the manual and control groups (mean difference=0.460, p=0.001). However, there was no significant difference between the digital and control groups (p=0.675). At the middle site, the digital group had a mean thickness of 7.00±0.223 mm, the manual group had 8.88±0.983 mm, and the control group had 7.00 mm. The pairwise comparisons revealed significant differences between the digital and manual groups (mean difference=-1.880, p=0.001) and between the manual and control groups (mean difference=1.880, p=0.001), but not between the digital and control groups (p=1.000). At the cervical site, the digital group showed a mean thickness of 9.06±0.134 mm, the manual group had 10.08±0.454 mm, and the control group had 9.00 mm. Significant differences were observed between the digital and manual groups (mean difference=-1.020, p=0.000) and between the manual and control groups (mean difference=1.080, p=0.000). However, there was no significant difference between the digital and control groups (p=0.936) (Figure 4).

**Table 1:** Pairwise comparison of the thickness values recorded for each group at three different sites. The p-value was derived using one-way ANOVA test and post hoc Tukey's analysis. * signifies a p-value of <0.05

Site	Group	Mean difference	Standard error	95% Confidence interval		p-value
				Lower	Upper	
Incisal	Digital vs. manual	-0.540	0.093	-0.788	-0.291	0.000*
	Digital vs. control	-0.080	0.093	-0.328	0.168	0.675
	Manual vs. control	0.460	0.093	0.2116	0.708	0.001*
Middle	Digital vs. manual	-1.880	0.368	-2.862	-0.897	0.001*
	Digital vs. control	0.000	0.368	-0.982	0.982	1.000
	Manual vs. control	1.880	0.368	0.897	2.862	0.001*
Cervical	Digital vs. manual	-1.020	0.173	-1.482	-0.557	0.000*
	Digital vs. control	0.060	0.173	-0.402	0.522	0.936
	Manual vs. control	1.080	0.173	0.617	1.542	0.000*

Vertical gap values

For vertical gap values, the study found no significant differences between the digital, manual, and control groups at any site. The pairwise comparisons indicated that the vertical gap differences were not statistically significant, with p-values of 0.688 (digital vs. manual), 0.642 (digital vs. control), and 0.997 (manual vs. control). This suggests that the vertical gaps recorded for each group were comparable across the different sites (Table 2).

**Table 2:** Pairwise comparison of the vertical gap values recorded for each group at three different sites

Groups	Mean difference	Standard error	95% Confidence interval		p-value
			Lower	Upper	
Digital vs. manual	0.148	0.178	-0.286	0.582	0.688
Digital vs. control	0.161	0.178	-0.273	0.595	0.642
Manual vs. control	0.013	0.178	-0.4210	0.447	0.997

Anatomical form values

Regarding anatomical form values, the digital group demonstrated superior accuracy and consistency compared to the control group. The digital group had a mean value of 1.00±0.00, while the control group had 2.93±0.798. The mean difference of -1.933 was highly significant (p=0.000). This indicates that the digital method produced more accurate and consistent anatomical forms compared to the manual method (Table 3).

**Table 3:** Pairwise comparison of the anatomical form values recorded for each group at three different sites * signifies a p-value of <0.05

Groups	Mean ± standard deviation	Standard error	Mean difference	95% Confidence Interval		T value	P value
				Lower	Upper		
Digital	1.00 ± 0.00	0.000	-1.933	0.206	-1.510	-9.374	0.000*
Control	2.93 ± 0.798	0.206	-1.933	0.206	-1.490		

## Discussion

The study compared the mean thickness and anatomical form accuracy of the digital, manual, and control groups at three sites. The digital group had lower thickness at the incisal (1.92±0.130 mm vs. 2.46±0.219 mm for manual, p=0.000), middle (7.00±0.223 mm vs. 8.88±0.983 mm for manual, p=0.001), and cervical sites (9.06±0.134 mm vs. 10.08±0.454 mm for manual, p=0.000). No significant differences were found in vertical gap values (p=0.688 to 0.997). These results demonstrate the digital method's enhanced precision and consistency, partially rejecting the null hypothesis.

In a study evaluating the marginal discrepancies of fixed partial denture pontics fabricated using computer-aided design and manufacturing (CAD-CAM) techniques, CAD-CAM methods demonstrated superior overall accuracy, except at the marginal surfaces [12]. Additively manufactured fixed partial denture pontics exhibited larger deviations, especially on the external and intaglio surfaces, which could necessitate additional clinical adjustments. While polyjet rapid prototyping showed slightly lower precision at the margins compared to bioprinting, the 2.3-micrometer difference was deemed clinically insignificant. Both intaglio surface deviations were under 30 micrometers, and marginal deviations were below 20 micrometers across all methods, suggesting comparable clinical fit [13]. A study supported these findings, noting similar accuracy for crown patterns [14]. Previous research indicates that CAD-CAM techniques often result in higher marginal gaps for additively manufactured patterns compared to subtractive methods [15]. A systematic review of CAD-CAM fabricated FPDs highlighted considerable variability in accuracy due to diverse experimental protocols, making direct comparisons challenging [16]. Limited clinical evidence directly contrasts the fit of CAD-CAM FPDs with conventionally fabricated ones. In a comparative study, frameworks produced via various digital workflows and conventional methods were assessed for fit using polyvinyl siloxane replicas and light microscopy. The results indicated that digital workflows generally achieved similar or superior marginal fit compared to conventional methods, demonstrating the efficacy of modern CAD-CAM techniques in maintaining clinical standards [17]. In a study, the CAD-CAM group exhibited a mean marginal gap of 26.80 μm, significantly smaller than the 38.83 μm gap observed in the conventional group. Both techniques achieved marginal gaps under 100 μm, which is considered acceptable for wax pattern fabrication of fixed partial dentures [18]. These findings are consistent with previously conducted studies that reported smaller gaps with CAD-CAM systems compared to conventional methods [19]. Contradictory results have been reported in other studies, where the conventional press technique also yielded lower marginal gaps compared to CAD-CAM methods [20, 21, 22]. Furthermore, previous research indicates no significant differences in the marginal fit of crowns fabricated by either conventional or CAD-CAM techniques [23].

Limitations of the study 

This study's limitations include the fabrication of all pontics by a single person and single cast, which may introduce variabilitythat is not representative of different scenarios. The controlled laboratory environment may not fully replicate the diverse conditions encountered in actual clinical settings, where technician skill and material handling can vary. Measurement precision using the stereo microscope and digital calipers may be subject to inherent inaccuracies. Additionally, the findings are specific to the CAD and CAM systems employed in this study, which may not encompass the full range of technologies available, potentially affecting the generalizability of the results.

## Conclusions

The study highlights the significant benefits of CAD-CAM technology over traditional manual methods in the fabrication of FPD pontics. CAD-CAM techniques resulted in pontics with reduced thickness and enhanced anatomical accuracy, demonstrating superior precision and consistency. Although vertical gap measurements were comparable across all methods, the digital approach achieved better overall prosthetic outcomes. These findings underscore the advantages of CAD-CAM in producing more accurate and reliable pontics, suggesting its potential to improve clinical practices and outcomes in prosthodontic rehabilitation.
